# Effectiveness of REAC neuro postural and neuro psycho physical optimization in improving peripheral vasospasm dysfunction: a case report

**DOI:** 10.3389/fmedt.2023.1198612

**Published:** 2023-04-28

**Authors:** Fabio Bechelli

**Affiliations:** Internal Medicine, International Scientific Society of Neuro Psycho Physical Optimization with REAC Technology, Brazilian Branch, São Paulo, Brazil

**Keywords:** peripheral vasospasm, depression, anxiety, stress, neuromodulation, neurostimulation, radio electric asymmetric conveyer, case report

## Abstract

This case report discusses an elderly male patient (86 years old), suffering from limb pain related to ulcers in the lower limbs resulting from peripheral arterial disease (PAD). Clinically evaluated with the aid of infrared thermal imaging before, during and after treatment, he was submitted to treatment with neuromodulation protocols with REAC Technology, Neuro Postural Optimization (NPO) and Neuropsychophysical Optimization (NPPO) in association with traditional treatments for PAD. It was followed clinically with the aid of infrared thermal imaging of the lower limbs before, during and after treatment. He had a clinical result with a significant reduction in pain and infrared thermal images with complete revascularization of both feet. Evidencing that the treatment of dysfunctional adaptive responses by managing psychological factors often associated with anxiety, depression and stress performed by the REAC NPO and NPPO protocols can be a useful intervention to improve symptoms of patients with lower limb pain and circulatory disturbances.

## 1. Introduction

Peripheral arterial disease (PAD) is a common disorder that affects the blood vessels in the lower limbs, causing reduced blood flow to the muscles and tissues. Vasospasm (1), a sudden constriction of the blood vessels, is a potential complication of PAD that can exacerbate symptoms and increase the risk of tissue damage and limb loss. While the pathophysiology of vasospasm is not fully understood, recent research suggests that psychological factors, such as depression, anxiety and stress, may play a role in its development (2, 3).

Anxiety and stress are known to activate the sympathetic nervous system, which regulates blood vessel tone and can cause vasoconstriction (4, 5). In individuals with PAD, this may lead to an increased risk of vasospasm and worsened symptoms (1).

Understanding the link between depression, anxiety and stress and vasospasm in PAD has important implications for the management of this condition (6). If depression, anxiety and stress are found to be significant risk factors for vasospasm (7), interventions targeting these factors may be a useful adjunct to traditional treatments for PAD, such as medication and lifestyle modifications.

In the present study, we report a case where Radio Electric Asymmetric Conveyer (REAC) technology was used to administer two treatments—Neuro Postural (NPO) and Neuro Psycho Physical Optimization (NPPO)—aimed at improving adaptive response, and consequently, mitigating depression, anxiety, and stress. The effectiveness of these treatments was evaluated in a patient with peripheral vasospasm of the lower limbs, a condition that had persisted for approximately four years.

The results demonstrated the effectiveness of NPO and NPPO in improving peripheral vasospasm of the lower limbs.

## 2. Case description

### 2.1. De-identified patient information

This case report presents the clinical findings of an 86-year-old male. Written informed consent was obtained from the patient.

### 2.2. Relevant physical examination and other clinical findings

The patient presented to our observation with chronic pain in bilateral wounds located in the medial perimalleolar region, which had not responded to previous wound dressings. The patient's pain was initially assessed using a 0–10 visual analog scale, and the reported pain level was between 8 and 9.

### 2.3. Relevant past interventions and their outcomes

The patient reported two previous operations: cardiac pacemaker implantation in 2014 for heart rhythm disturbances and coronary stent implantation surgery in 2016.

## 3. Diagnostic assessment

**Figure 1:**
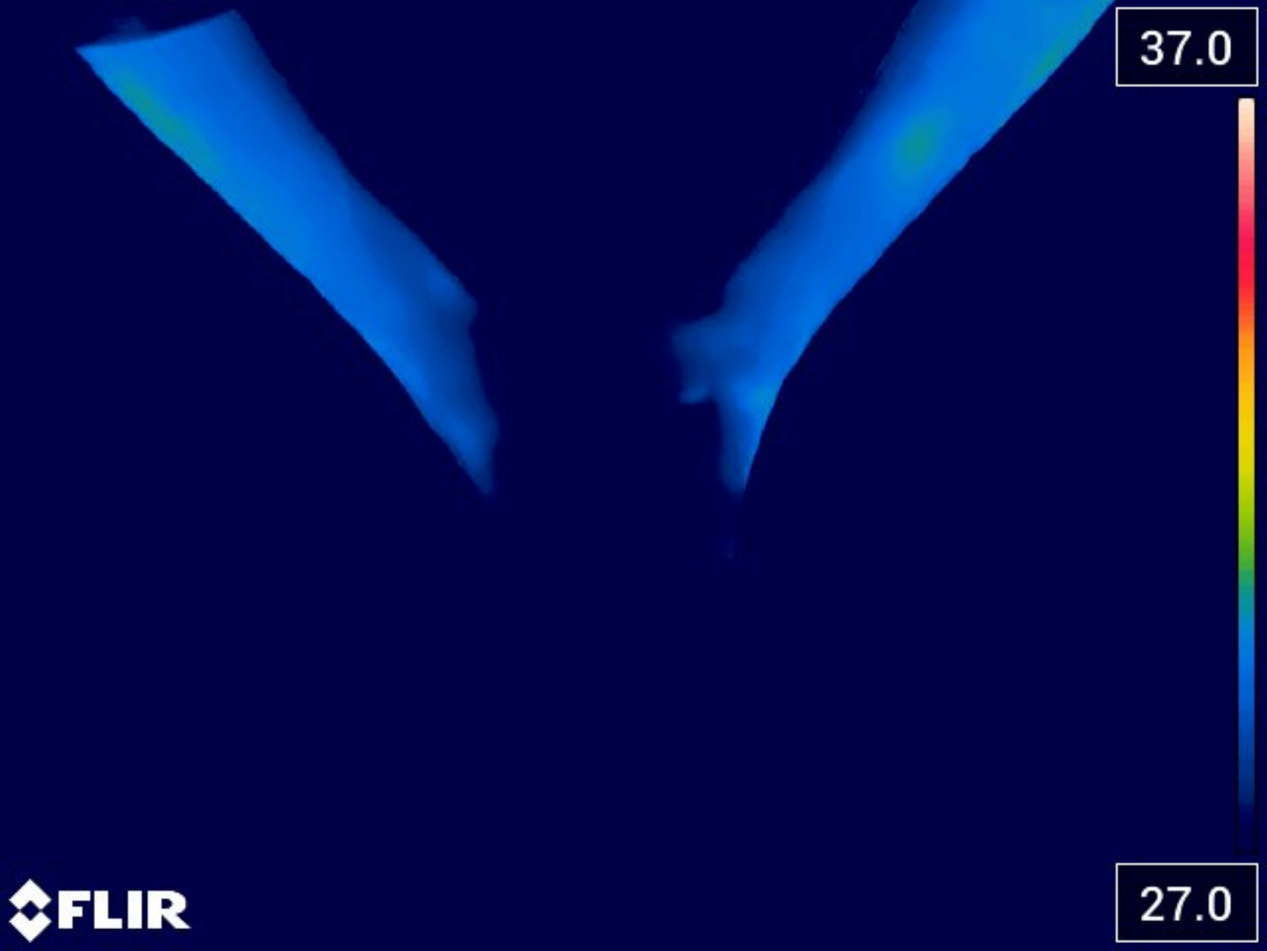
The thermal image obtained during the first visit shows the state of vasospasm in the lower third of the legs and feet, which results in a lack of thermal visualization of the affected areas.

During the initial assessment, we aimed to analyze the vascular perfusion in the perimalleolar area. To accomplish this, we employed a FLIR thermal imaging camera with a sensitivity range set between 27 and 37 degrees Celsius. The image obtained from the diagnostic examination revealed a distinctive and concerning circulatory amputation pattern, which appeared to originate from the lower third of the legs (see Figure 1).

This visual representation highlights the severity and complexity of the circulatory issue, which may potentially lead to further complications if left untreated.

## 4. Therapeutic intervention

**Figure 2:**
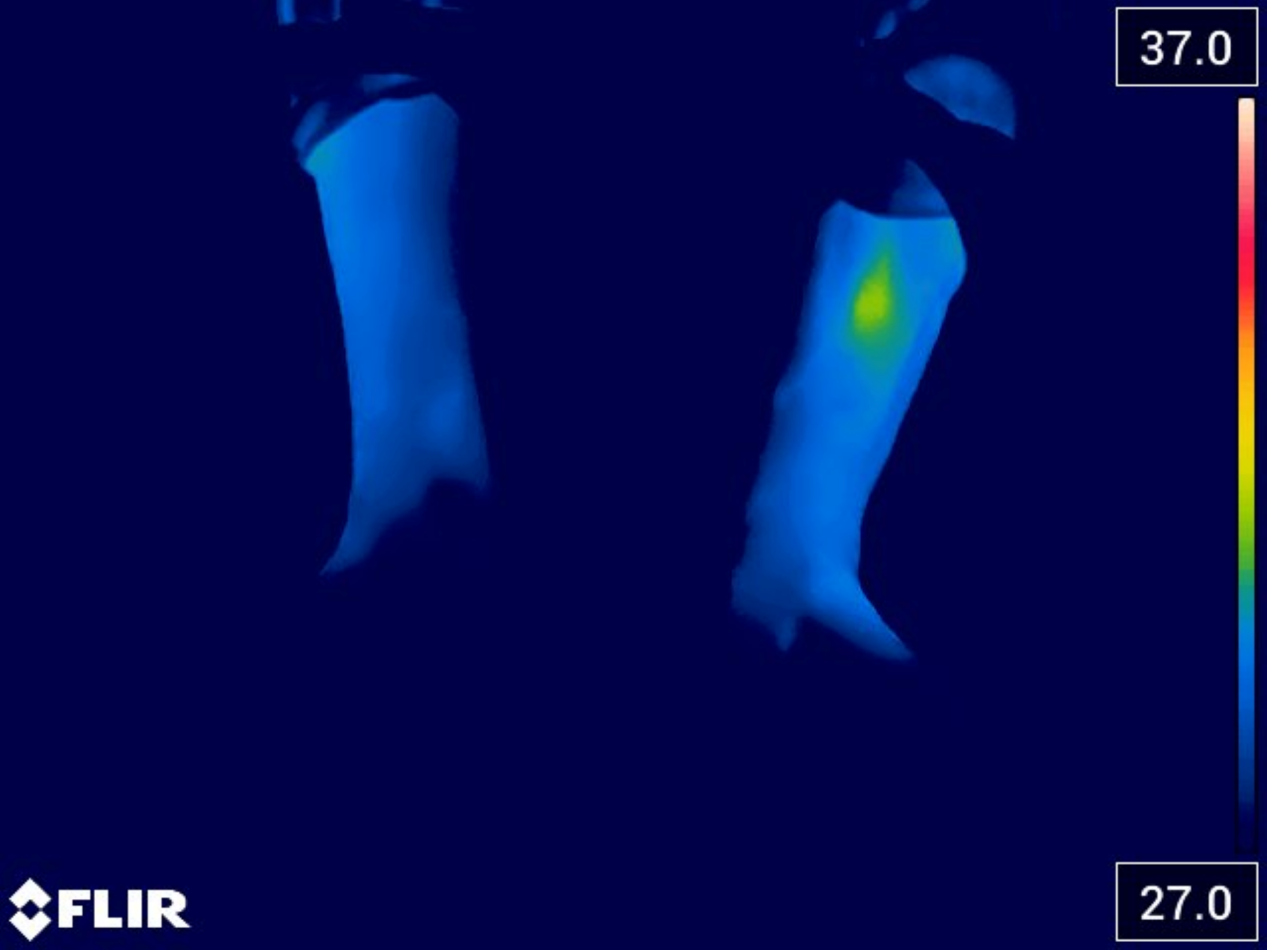
The thermal image acquired after the administration of REAC NPO treatment shows an initial reduction in the vasospasm state of the lower third of the legs and the left foot.

In order to improve the thermal imaging camera analysis and the patient's symptomatic picture, a single REAC NPO treatment session was administered. This treatment produced an immediate and sustained response, resulting in the resolution of functional dysmetria and the initiation of a cascade of neurovegetative events that could contribute to the amelioration of vasospasm (Figure 2).

The acquisition of a thermal image immediately after administering the REAC NPO treatment indicates an initial improvement, characterized by the reappearance of vascularization in the left perimalleolar area. This finding suggests the presence of a dysfunctional component in the thermo-vascular amputation and highlights the potential of the REAC NPO treatment to stimulate the initial restoration of blood flow and tissue oxygenation, a crucial factor in wound healing.

After undergoing REAC NPO treatment, a follow-up pain assessment was conducted, which revealed an improvement from the initial score of 8–9 to a score of 7, which was recorded.

**Figure 3:**
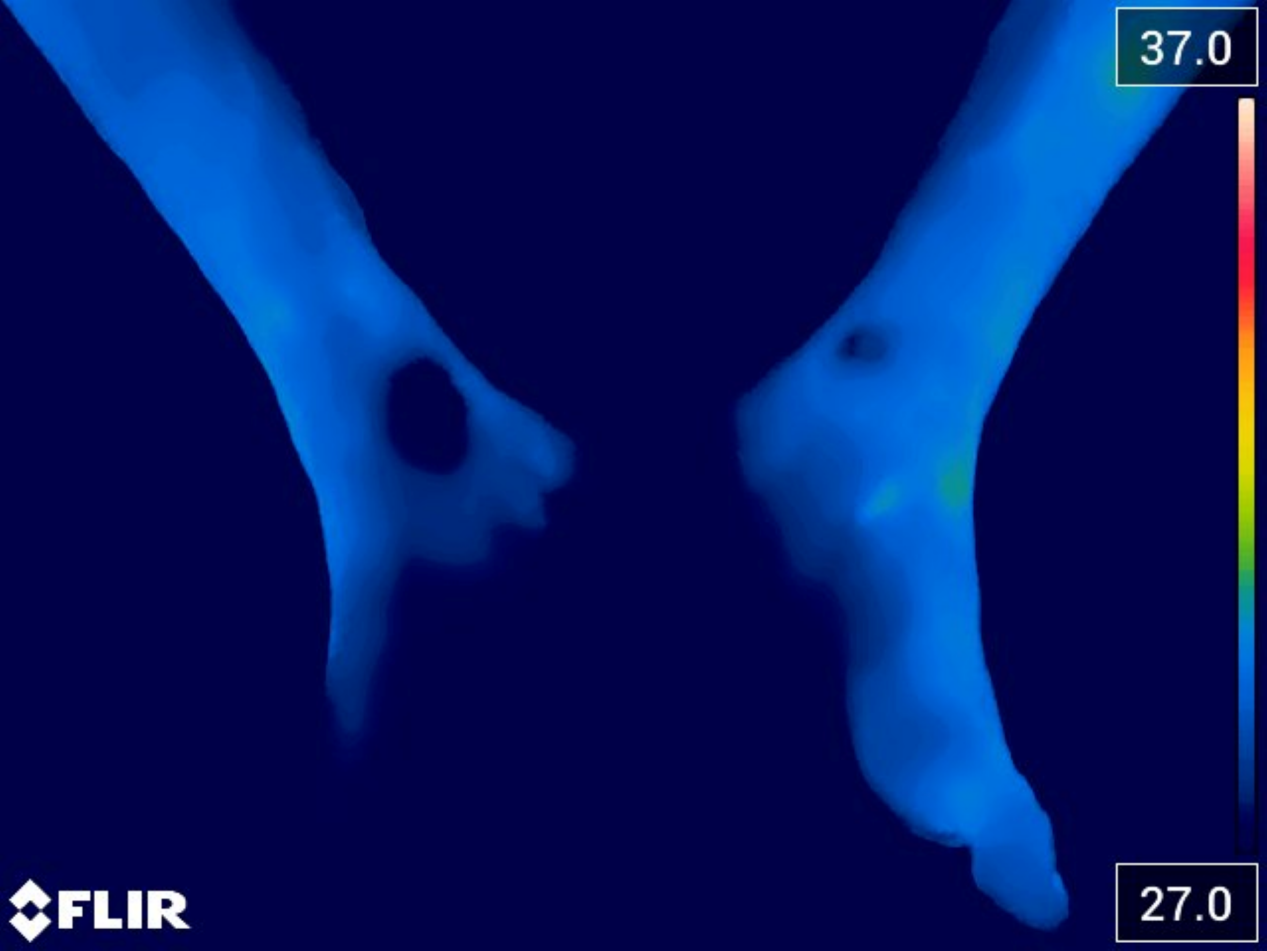
Thermal image acquired one week after administration of REAC NPO treatment. The image highlights a further improvement of the bilateral vasospasm in the two limbs.

One week after undergoing the REAC NPO treatment, the patient underwent a follow-up check-up and a new thermal imaging was acquired, demonstrating further improvement compared to the initial images (Figure 3).

In addition, pain assessment was conducted during the follow-up, and it was observed that the pain score had improved from 7 to 5–6.

After observing the positive outcomes resulting from the initial neurovegetative modulation induced by the REAC NPO treatment, we recommended that the patient continue with the NPPO treatment. The NPPO treatment is specifically designed to enhance mood and alleviate behavioral disorders including depression, anxiety, and stress related disorders.

The REAC NPPO is a neurobiological modulation treatment aimed at improving the ability to respond to environmental interaction from a neurological, psychological, and physical point of view (8–12). Each treatment cycle consists of 18 sessions, to be administered from one to a maximum of four sessions per day. Each treatment session lasts approximately three seconds. In this scenario, the patient underwent two weekly treatments of REAC NPPO spaced one hour apart.

The REAC treatment parameters are predetermined by the manufacturer and cannot be altered by the operator. To administer REAC treatments, a BENE 110 device (ASMED, Florence, Italy), was used.

## 5. Follow-up and outcomes

**Figure 4:**
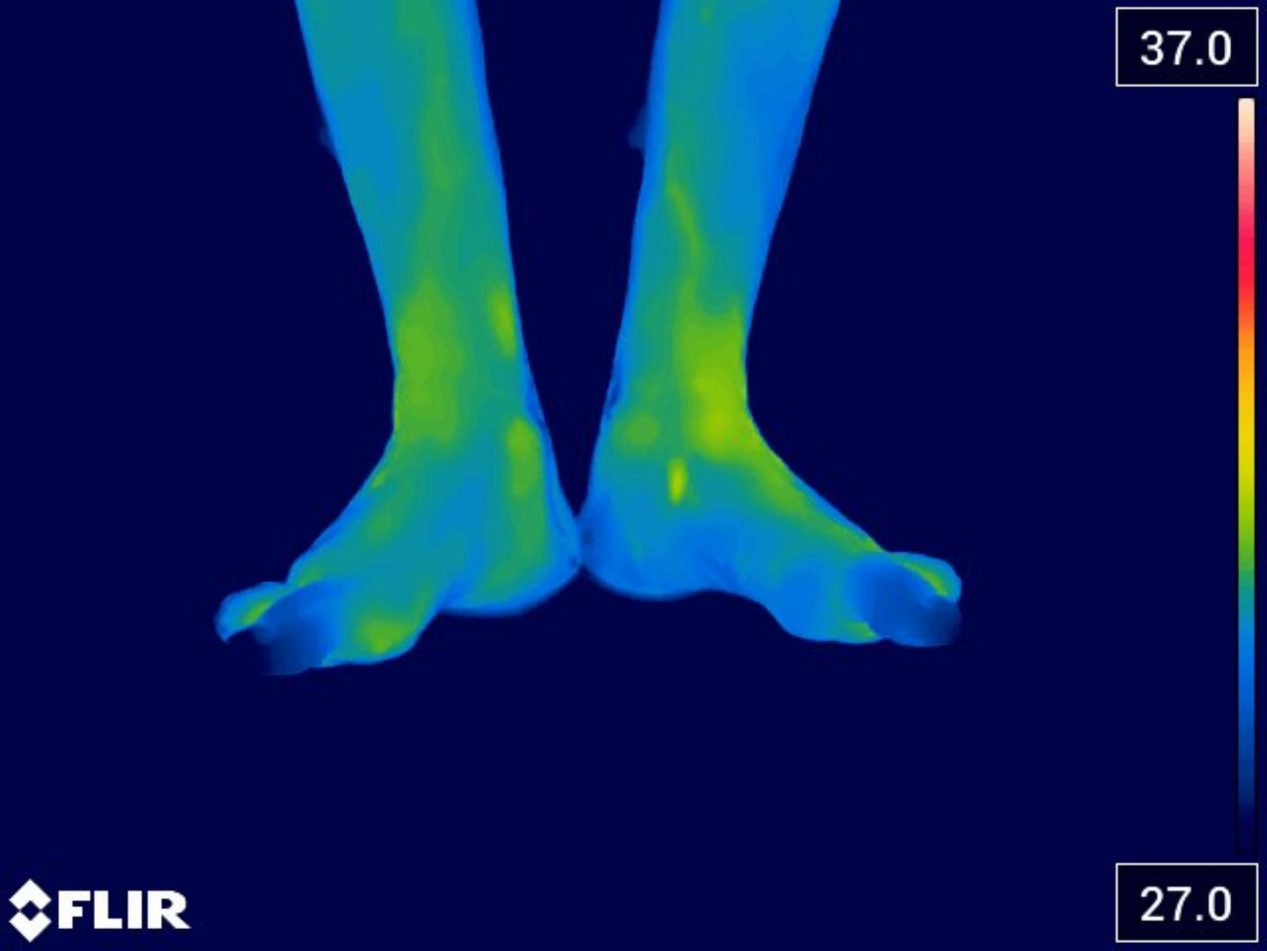
Thermal image acquired the week after the end of 18 sessions of REAC NPPO, which reveals complete revascularization of both feet.

After completing 18 sessions of REAC NPPO, the patient underwent a follow-up assessment the following week. Thermal imaging was performed and revealed complete revascularization of both feet (Figure 4).

In the final follow-up, a new evaluation of the patient's perceived pain was conducted, revealing that while the pain had not completely disappeared, it was rated between 1 and 2 on the evaluation scale.

## 6. Discussion

There is a growing body of scientific evidence indicating that depression, anxiety, and stress can contribute to the development and exacerbation of vasospasm and pain (2, 13). Vasospasm is a condition characterized by the sudden constriction of blood vessels, which can cause reduced blood flow to vital organs and tissues. Pain is often associated with vasospasm, as the reduced blood flow can cause tissue damage and inflammation (14, 15).

Depression, anxiety, and stress are known to increase the levels of stress hormones such as cortisol, which can promote inflammation and blood vessel constriction (16). These changes can lead to the development of vasospasm and pain (17). Additionally, depression and anxiety are associated with alterations in pain perception and processing in the brain, which can lead to increased pain sensitivity and intensity (18, 19).

Also, stress can trigger vasospasm and increase pain sensitivity through a variety of mechanisms, including activation of the sympathetic nervous system and release of pro-inflammatory cytokines.

In summary, depression, anxiety, and stress can contribute to the development and exacerbation of vasospasm and pain through various mechanisms.

Drawing on these considerations, it is possible to appreciate how the REAC NPO and NPPO neuromodulation treatments, which target dysfunctional adaptive responses frequently associated with depression, anxiety, and stress, have demonstrated efficacy in reducing vasospasm and restoring distal circulation in the lower extremities.

## 7. Patient perspective

After analyzing the obtained results, it can be concluded that the patient's chances of maintaining restoration of distal circulation in the lower limbs are positive, particularly if the patient continues to manage depression, anxiety, and stress on a regular basis. Additionally, the patient may benefit from REAC neuromodulation treatments in their management of these psychological factors.

## 8. Conclusion

In conclusion, the lack of improvement in pain and distal circulation of the lower limbs with conventional physiotherapy treatments underscores the importance of addressing the neurovegetative dysfunctional component related to depression, anxiety and stress in patients. Current scientific evidence supports the use of neuromodulation treatments, such as REAC NPO and NPPO, as useful interventions for improving such symptomatology in patients with lower limb pain and circulatory disturbances.

## Data Availability

The original contributions presented in the study are included in the article, further inquiries can be directed to the corresponding author.
